# Functional interaction of melatonin with gasotransmitters and ROS in plant adaptation to abiotic stresses

**DOI:** 10.3389/fpls.2024.1505874

**Published:** 2024-12-12

**Authors:** Yuriy E. Kolupaev, Alla Yemets, Tetiana O. Yastreb, Yaroslav Blume

**Affiliations:** ^1^ Yuriev Plant Production Institute, National Academy of Agrarian Sciences of Ukraine, Kharkiv, Ukraine; ^2^ Institute of Food Biotechnology and Genomics, National Academy of Sciences of Ukraine, Kyiv, Ukraine

**Keywords:** melatonin, ROS, nitric oxide, hydrogen sulfide, carbon monoxide, cell signalling, protein post-translational modifications, abiotic stress

## Abstract

Melatonin is considered a multifunctional stress metabolite and a novel plant hormone affecting seed germination, root architecture, circadian rhythms, leaf senescence, and fruit ripening. Melatonin functions related to plant adaptation to stress stimuli of various natures are considered especially important. One of the key components of melatonin’s stress-protective action is its ability to neutralise reactive oxygen species (ROS) and reactive nitrogen species directly. However, many of its effects are related to its involvement in the signalling network of plant cells and its influence on the expression of a large number of genes important for adaptation to adverse factors. Insights into the functional relationships of melatonin with gasotransmitters (GT) – gaseous molecules performing signalling functions – are still emerging. This review has analysed and summarised the experimental data that testify to the participation of the main GTs – nitric oxide, hydrogen sulfide, and carbon monoxide – in the implementation of the protective effect of melatonin when plants are exposed to abiotic stimuli of various nature. In addition, modulation by melatonin of one of the most important components in the action of GTs and ROS – post-translational modifications of proteins and the influence of ROS and GTs on melatonin synthesis in plants under stress conditions and the specific physiological effects of exogenous melatonin and GTs have been reviewed. Finally, the prospects of the GTs’ practical application to achieve synergistic stress-protective effects on plants have been considered.

## Introduction

World agricultural statistics show that abiotic stresses have become the main factors limiting crop production in recent decades. They are responsible for more than a 50% reduction in the productivity of most crops (Kul et al., 2019). Climate change trends increase the importance of research aimed at developing new biotechnological tools to improve plant resistance. Enhancing the resistance of plants is actively pursued combining selection-genetic (traditional methods of selection and new means of genetic engineering) and physiological approaches. The latter includes a wide range of plant hormones, their synthetic analogues, as well as signalling mediators and stress metabolites, which are often collectively referred to as ‘bioregulators’ (Srivastava et al., 2016; Zulfiqar and Ashraf, 2021). Expanding the knowledge of compounds involved in the regulation of plant adaptive responses opens new opportunities both for the use of effective exogenous treatments of plants with new physiologically active substances and the control of their synthesis in plants by genome editing methods (Raza et al., 2022).

The regulation of adaptive responses and plant growth under stress conditions is mediated by a highly complex network of molecules. These include plant hormones (auxins, gibberellic acid, cytokinins, abscisic acid (ABA), salicylic acid, jasmonates, ethylene) (Ali et al., 2024), key signalling mediators (calcium ions, reactive oxygen species (ROS), nitric oxide, cyclic nucleotides) (Srivastava et al., 2016; Roy Chowdhury et al., 2019), and several compounds that combine the properties of stress metabolites and plant hormones and/or signalling network participants. The last group of compounds is currently the subject of the most in-depth research, resulting in the accumulation of a huge amount of experimental data.

The main representatives of this group of compounds turned out to be the so-called plant neurotransmitters, substances that act as mediators of nerve impulse transmission in animals (melatonin, serotonin, dopamine, acetylcholine, and gamma-aminobutyric acid) (Akula and Mukherjee, 2020). Within this group of compounds, the greatest research interest has been focused on melatonin (N-acetyl-5-methoxytryptamine) (Colombage et al., 2023; Taboada et al., 2023). For instance, an analysis of publications in the Web of Science™ database revealed that melatonin was the fourth most studied exogenous substance for mitigating plant stress caused by the most common factor, drought, over the past 24 years, preceded only by the major plant stress hormone ABA, signalling mediator hydrogen peroxide, and antioxidant glutathione (Feng et al., 2024). Owing to its low toxicity and environmental safety, melatonin (Pardo-Hernández et al., 2021) is regarded as a promising natural biostimulant for sustainable crop production under adverse environmental conditions (Manzoor et al., 2023).

The impact of melatonin on various stages of plant ontogenesis, including circadian rhythms, seed germination, root development, leaf senescence, flowering, seed formation, and fruit maturation, has been demonstrated (Pan et al., 2023; Zhao and Hu, 2023; Wang et al., 2024). Numerous data have also been accumulated on increasing the resistance of plants to abiotic stresses of various types under the influence of exogenous melatonin. These data are the subject not only of analytical reviews but also of meta-analyses (Agathokleous et al., 2021; Wang et al., 2022b; Plokhovska et al., 2023).

Melatonin is involved in the complex signalling network of plant cells through several mechanisms. One of the key components of this network is gasotransmitters (GTs) (Yao et al., 2019; Kolupaev et al., 2022). This term unites small gaseous molecules that are synthesised by living organisms and perform signalling functions. In contrast to hormones, their effects are not receptor-dependent; rather, they act on multiple intracellular targets of protein nature (Dey et al., 2024). Nitric oxide (NO), carbon monoxide (CO) and hydrogen sulfide (H_2_S) are considered to be the main GTs of plant cells (Yao et al., 2019; Kolupaev et al., 2022; Dey et al., 2024).

Post-translational modification (PTM) of protein thiol groups is an important regulatory mechanism of GTs such as NO and H_2_S (Zhang and Liao, 2019; Aroca et al., 2021; Martí-Guillén et al., 2022). The same groups are easily oxidised by ROS, especially by H_2_O_2_ (Valderrama et al., 2019; Xuan et al., 2020). There are reasons to believe that melatonin, as a redox-active molecule, can affect protein PTM processes induced by ROS and GTs. However, despite the intensive accumulation of data on the involvement of both melatonin and GTs in the regulation of plant adaptive responses, the data on their functional interaction remain poorly systematised (Wang et al., 2022a). On the other hand, data on the relationship between melatonin and two other important GTs, H_2_S and CO, in the formation of adaptive responses in plants are still limited and have not been analysed from the point of view of the functioning of the signalling network as a whole. Taking into account the above mentioned, this paper presents a review and analysis of data on the mechanisms of participation of the main GTs in the realisation of the protective effects of melatonin under the influence of abiotic stresses on plants.

## A brief summary of melatonin synthesis and metabolism in plant cells

**Figure 1:**
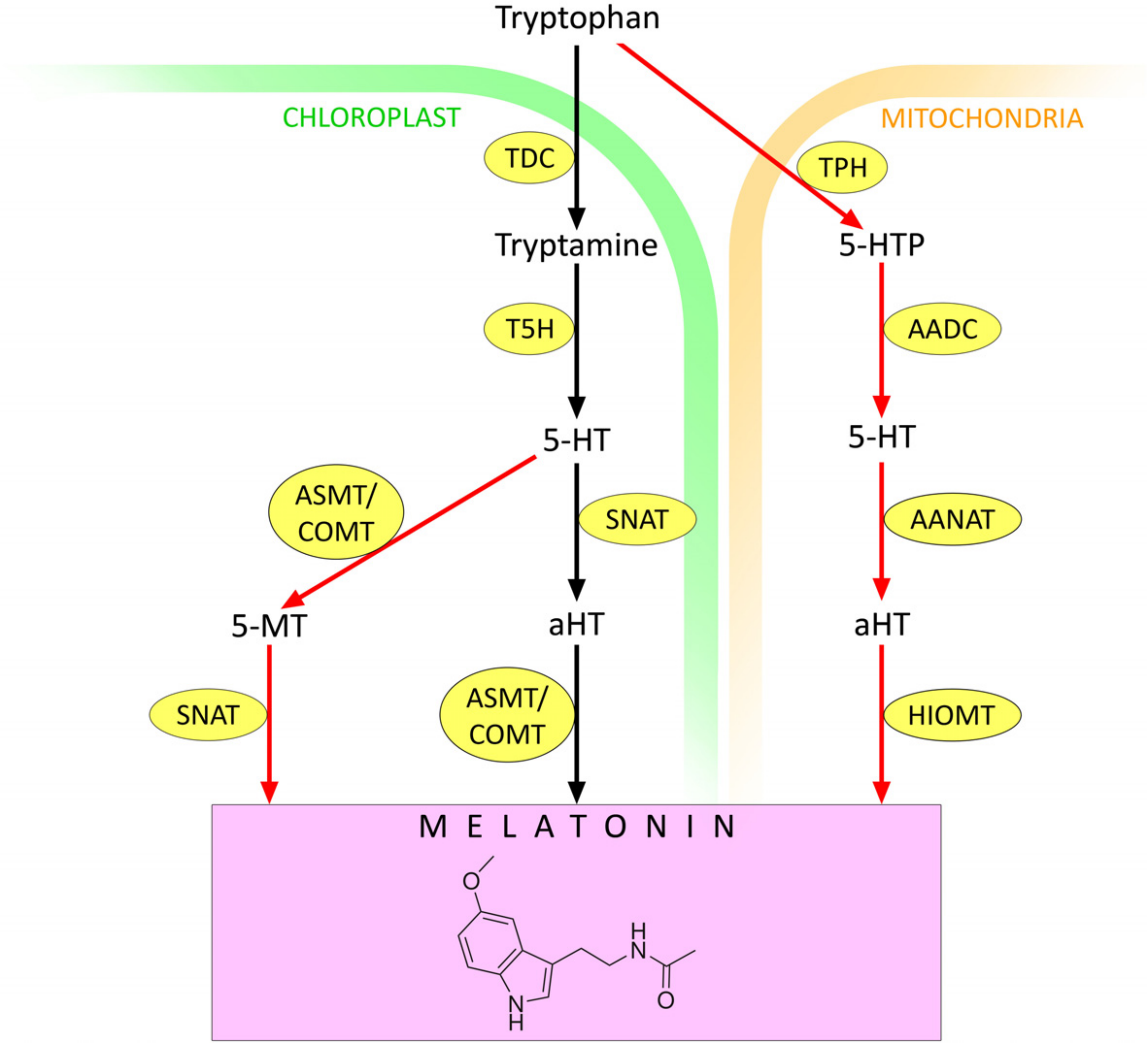
Melatonin synthesis in plants. 5-HT – 5-hydroxytryptamine (serotonin); 5-MT – 5-methoxytryptamine; AADC – aromatic amino acid decarboxylase; AANAT – arylalkylamine N-acetyltransferase (arylamine N-acetyltransferase); aHT – N-acetyl 5-hydroxytryptamine; ASMT – N-acetylserotonin O-methyltransferase; COMT – caffeic acid O-methyltransferase; HIOMT – hydroxyindole O-methyltransferase (N-acetylserotonin O-methyltransferase); SNAT – serotonin N-acetyltransferase; T5H – tryptamine 5-hydroxylase; TDC – tryptophan decarboxylase; TPH – tryptophan hydroxylase. Red arrows indicate melatonin synthesis pathways that are activated under stressful conditions. Other explanations in the text.

In plants, melatonin is thought to be synthesised predominantly in chloroplasts (Zeng et al., 2022) (**Figure 1**). First, tryptophan decarboxylase (TDC) converts tryptophan to tryptamine by removing the carboxyl group. Then, under the influence of tryptamine 5-hydroxylase (T5H), tryptamine is hydroxylated to serotonin (5-hydroxytryptamine, 5-HT). The latter is converted to melatonin in two steps by serotonin N-acetyltransferase (SNAT) and N-acetylserotonin O-methyltransferase (ASMT, also known as caffeic acid O-methyltransferase, COMT) (Rehaman et al., 2021). However, the order of the transformations can vary depending on environmental conditions (Zeng et al., 2022). Under standard conditions, SNAT-catalysed acetylation of serotonin to N-acetyl 5-hydroxytryptamine (aHT) occurs first, followed by its O-methylation by ASMT/COMT, leading to melatonin (Ye et al., 2019). In contrast, abiotic stress induces the expression of different isoforms of ASMT/COMT so that serotonin is first O-methylated to 5-methoxytryptamine (5-MT). Subsequently, 5-MT is acetylated by SNAT to form melatonin (Tan and Reiter, 2020).

In the mitochondria, tryptophan can be converted to 5-hydroxytryptophan by the action of tryptophan hydroxylase (TPH). Then 5-HT is formed under the action of aromatic amino acid decarboxylase (AADC). 5-HT is converted to aHT by arylalkylamine N-acetyltransferase (AANAT), also known as arylamine N-acetyltransferase. Finally, aHT is converted to melatonin by the action of hydroxyindole O-methyltransferase (HIOMT), also known as N-acetylserotonin O-methyltransferase (Zeng et al., 2022) (**Figure 1**). There is evidence that the mitochondrial contribution to melatonin synthesis increases under stress conditions (Zeng et al., 2022). Therefore, depending on the environment, both the contribution of different chloroplast pathways to melatonin synthesis and the ratio of melatonin synthesis in chloroplasts and mitochondria may change.

To date, a rather extensive phenomenology of melatonin synthesis enhancement in plants in response to stress stimuli of different natures has been accumulated. For example, an almost twofold increase in melatonin content was observed in wheat and rice plant organs in response to heating (Byeon and Back, 2014; Buttar et al., 2020). Similar effects at high temperatures were found in *Arabidopsis* (Shi et al., 2015) and tomato plants (Xu et al., 2016). Under salt, osmotic, and heat stress, an increase in endogenous melatonin levels was detected in grapes, barley, and lupin (Hassan et al., 2022).

The mechanisms of melatonin perception by plant cells have not been fully studied. Plant cells, similar to animal cells, contain three subunits of the G-protein complex (Gα, Gβ, and Gγ) and several putative G-protein-coupled receptors (GPCR) (Cannon and Chapman, 2021). In plants, CAND2 and CAND7 proteins have been found to interact with GPCR (Jin et al., 2012). It is the CAND2 protein that has been proposed as the phytomelatonin receptor1 (PMTR1) in 2018 (Wei et al., 2018). Experimental data have been obtained that implicate the CAND2/PMTR1 protein in the activation of Ca^2+^ influx and K^+^ efflux during melatonin-induced stomatal closure. The *Arabidopsis AtCand2* mutant, in contrast to wild-type plants, did not respond to melatonin treatment with stomatal closure (Wei et al., 2018). However, confocal microscopy data indicate that the CAND2 protein is localised in cytosol (Lee and Back, 2020). It is therefore, suggested that CAND2 might not be the G-protein that mediates melatonin-induced effects. Recently, receptor-like kinases (RLKs) have been considered as alternative candidates for melatonin receptors in plants (Back, 2021). *Arabidopsis* is known to have more than 600 RLK homologs. Since the MAP kinase cascade was activated in response to melatonin action, it is likely that one of the 600 RLKs in *Arabidopsis* functions as a melatonin receptor (Back, 2021). Nevertheless, recent experimental evidence points to a role for PMTR1 in melatonin perception and action in drought, salinity, and pathogen response in alfalfa, tobacco, and maize (Khan et al., 2023). For example, osmotic stress was shown to significantly increase *PMTR1* transcript levels in plants in a mannitol dose-dependent manner (Wang et al., 2021). In addition, it was recently shown that overexpression of the *MePMTR1* gene isolated from the tropical cassava plant (*Manihot esculenta* Crantz) in *Arabidopsis* plants resulted in an increased resistance to dark-induced senescence compared to the wild type (Cheng et al., 2024). However, the presence and cellular localisation of melatonin receptors in higher plants still need further investigation (Corpas et al., 2022a).

## Functional interaction of melatonin with ROS

ROS are essential signalling molecules that play diverse roles in the rapid response of plants to environmental stimuli (Ahammed et al., 2024). However, ROS generally act as signalling mediators when their levels are increased in individual cell compartments in a short-term and cell-controlled manner (Kolupaev et al., 2019; Dvořák et al., 2021; Li and Kim, 2022). Excessive production of ROS can cause programmed cell death, and large-scale stochastic ROS production can lead to uncontrolled destructive cell changes (Kacperska, 2004; Hasanuzzaman et al., 2020).

ROS are generated in one-, two-, and three-electron oxygen reduction reactions as a result of spontaneous and enzymatic oxidation of various substrates, as well as in photoinduced reactions (Sachdev et al., 2021). In higher plant cells, the primary sources of ROS are the electron transfer chains present in chloroplasts and mitochondria, but various ROS-generating enzymes are present in subcellular compartments (Taboada et al., 2023).

### ROS generation in chloroplasts

Photosynthesis is one of the major sources of ROS in green plant cells. Superoxide anion radicals O_2_^•−^ are generated by photosystem I and II electron transport chain (ETC) function (Li and Kim, 2022). The photoinduced generation of ROS mainly depends on environmental conditions and the physiological state of photosynthetic apparatus (Foyer and Shigeoka, 2011). When CO_2_ fixation is limited under various stressors (e.g., drought, salinity, and high temperature), the NADPH pool is only slightly consumed, resulting in electron “leakage” from ferredoxin to molecular oxygen to form O_2_^•−^. Superoxide anion radicals formed in chloroplasts are readily converted to the more stable ROS, hydrogen peroxide H_2_O_2_, under the influence of superoxide dismutase (SOD).

Singlet oxygen is also formed in chloroplasts, primarily as a consequence of the transition of chlorophyll P_680_ to the triplet state within the reaction centre of photosystem II and/or the light-harvesting complex. The likelihood of singlet oxygen formation, as well as other ROS, increases when the ETC is over-reduced, which is characteristic of stressful conditions (Li and Kim, 2022).

### ROS generation in mitochondria

Mitochondria, similar to chloroplasts, contain a large number of electron carriers. Their inadvertent interaction with molecular oxygen can lead to a one-electron reduction of O_2_ to O_2_^•−^. The main electron leakage sites in plants, as in animals, are considered to be complexes I and III of the ETC (Cvetkovska and Vanlerberghe, 2013). However, the ETC of plant mitochondria also involves an alternative electron transport pathway. It includes two molecules of NAD(P)H dehydrogenase on the outer and inner sides of the inner mitochondrial membrane, alternative oxidase, and an uncoupling protein, UCP, which limit ROS generation (Sachdev et al., 2021). The functions of these proteins are enhanced under stressful conditions.

### ROS synthesis in peroxisomes

Peroxisomes are also among the compartments that generate significant amounts of ROS in processes such as photorespiration and β-oxidation of fatty acids mediated by acyl-CoA oxidase (Sachdev et al., 2021). ROS generation in peroxisomes may also be related to the activities of flavin oxidase, urate oxidase, xanthine oxidase and other enzymes (Hasanuzzaman et al., 2020). Under abiotic stress, photorespiration is initiated in the chloroplast because of the limited availability of CO_2_ and increased solubility of O_2_, which competitively accelerates the oxygenation of ribulose-1,5-biphosphate to form glycolate. The latter is exported to peroxisomes, where it is oxidized by glycolate oxidase to form H_2_O_2_ (del Río et al., 2003; Das and Roychoudhury, 2014).

### ROS generation in the apoplast

The apoplast is the site of generation of a large amount of ROS, which may be a component of cell signalling. For example, NADPH oxidase, known as the Respiratory Burst Oxidase Homologs (RBOH), is localised on the plasma membrane (Marino et al., 2012). This enzyme complex reduces molecular oxygen to form superoxide anion radical. *Arabidopsis* genome contains 10 members of the RBOH membrane-bound (catalytic) subunit gene family, and the involvement of some of them in signalling processes has been confirmed by molecular genetic methods (Torres et al., 2002; Hasanuzzaman et al., 2020). A significant pool of signalling ROS appears to be also generated in the apoplast by the oxidase activity of class III heme peroxidases (Gautam et al., 2017).

### Involvement of melatonin in ROS regulation

As shown previously, melatonin affects cellular content of ROS as a direct antioxidant and as a probable participant in the signalling network and inducer of other components of the antioxidant system (Ahammed et al., 2019).

Melatonin can directly interact with ROS such as ^•^OH, H_2_O_2_, and ^1^O_2_ (Galano and Reiter, 2018). Melatonin has been reported to be more active than glutathione and mannitol in binding hydroxyl radicals (Arnao and Hernández-Ruiz, 2019). The chemical mechanisms of melatonin interaction with different ROS and their corresponding constants have been described in several reviews (Galano and Reiter, 2018; Arnao and Hernández-Ruiz, 2019).

The catabolism of melatonin by ROS is one of the mechanisms by which melatonin exerts its direct antioxidant action. In addition, melatonin metabolites also possess direct and indirect antioxidant properties (Taboada et al., 2023).

**Figure 2:**
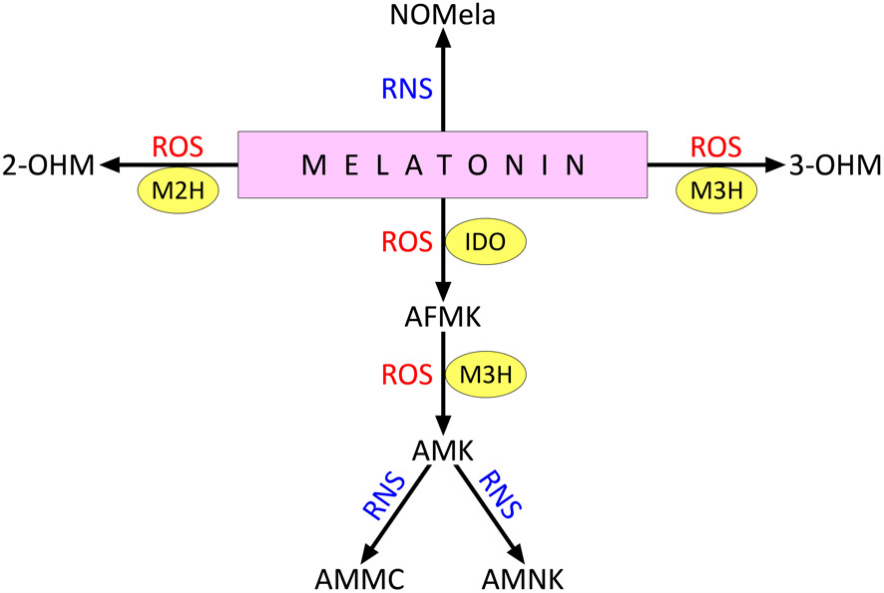
Direct interaction of melatonin with ROS and RNS. 2-OHM, 2-hydroxymelatonin; 3-OHM, 3-hydroxymelatonin; AFMK , cyclic N1-acetyl-N 2-formyl-5-methoxykynuramine; AMK , N1-acetyl-5-methoxykynuramine; AMMC , acetamidomethyl-6-methoxycinnolinone; AMNK , N1-acetyl-5-methoxy-3-nitrokynuramine; IDO , indoleamine 2,3-dioxygenase; M2H , melatonin 2-hydroxylase; M3H , melatonin 3-hydroxylase; NOMela , N-nitrosomelatonin; ROS , reactive oxygen species; RNS , reactive nitrogen species. Other explanations in the text.

Melatonin catabolism can occur with or without enzyme involvement (Back, 2021; Zeng et al., 2022). One pathway involves the conversion of melatonin to cyclic N^1^-acetyl-N ^2^-formyl-5-methoxykynuramine (AFMK) by indoleamine 2,3-dioxygenase (IDO) (Wang et al., 2024) (**Figure 2**). AFMK is later converted to N^1^-acetyl-5-methoxykynuramine (AMK) by the action of melatonin 3-hydroxylase (M3H). Melatonin can also be converted to 2-hydroxymelatonin (2-OHM) and 3-hydroxymelatonin (3-OHM) by interaction with ROS; these processes involve M2H and M3H, respectively (Back, 2021). 2-OHM and 3-OHM are the predominant melatonin metabolites in plants (Back, 2021; Khan et al., 2023). In addition, the formation of 4-hydroxymelatonin and 6-hydroxymelatonin is possible under the influence of radical ROS, the latter being more characteristic of melatonin degradation in animals (Mannino et al., 2021).

Melatonin also interacts with RNS, particularly when it reacts with peroxynitrite or nitrogen dioxide to form N-nitrosomelatonin (NOMela) (**Figure 2**). The possible physiological functions of this compound are discussed below in the section on the functional interactions between melatonin and NO. In addition, AMK formed by the interaction of AMFK with ROS can react with RNS to form 3-acetamidomethyl-6-methoxycinnolinone (AMMC) or N^1^-acetyl-5-methoxy-3-nitrokynuramine (AMNK) (Mannino et al., 2021).

The melatonin hydroxymetabolites 2-OHM and 3-OHM are present in plant cells at much higher levels than melatonin itself. For example, it has been reported that in an intact rice leaf, the content of 3-OHM and 2-OHM exceeds the content of melatonin by 300 and 100 times, respectively (Byeon et al., 2015; Taboada et al., 2023). Even higher concentrations of melatonin hydroxymetabolites were recorded in rice under stress conditions, particularly under cadmium influence and salinity stress (Lee et al., 2017; Choi and Back, 2019).

The hydroxymetabolite 3-OHM, formed from melatonin as a result of M3H activity, has intrinsic antioxidant activity targeting hydroxyl and hydroperoxyl (^•^OOH) radicals (Tan et al., 2014). In reaction with 1,1-diphenyl-2-picrylhydrazyl, 3-OHM was shown to have 15 times more antioxidant activity than melatonin (Lee and Back, 2022).

**Figure 3:**
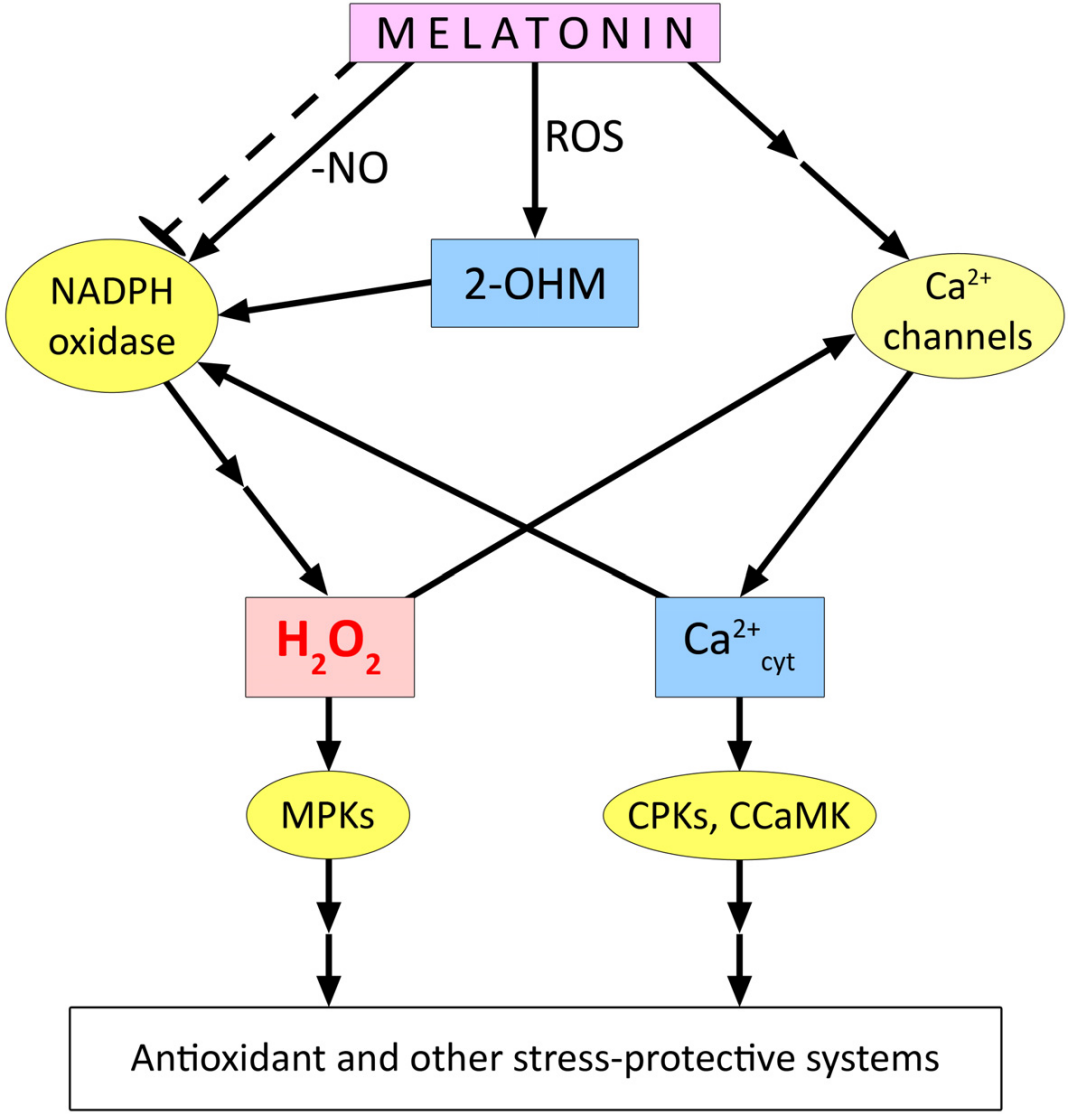
Functional interaction of melatonin with ROS in plant cells. 2-OHM – 2-hydroxymelatonin; CPKs, Ca^2+^-dependent protein kinases; CCaMK, Ca^2+^/calmodulin-dependent protein kinase; ROS, reactive oxygen species. The dashed arrow with blunt end indicates the possible inhibitory effect of melatonin on the expression of genes encoding certain molecular forms of NADPH oxidase. Other explanations in the text.

The indirect effect of melatonin and its derivatives on the redox homeostasis of plant cells is even more complex. Several studies have reported increased gene expression and activity of antioxidant enzymes in plants of different taxonomic groups under normal and stress conditions (Magri and Petriccione, 2022; Zhang et al., 2022; Kolupaev et al., 2023a; Taboada et al., 2023). Such effects suggest the formation of ROS signalling under the influence of melatonin, which induces the antioxidant system (**Figure 3**). Indeed, some evidence has been obtained for the involvement of ROS generated by NADPH oxidase in the realisation of the stress-protective effect of melatonin (Gong et al., 2017; Wei et al., 2018; Sun et al., 2021). One of the mechanisms for the increase in NADPH oxidase activity under the action of melatonin may be related to the decrease in S-nitrosation of cysteine residues in its catalytic subunit (Gong et al., 2017). NO is known to participate in the S-nitrosation reaction of NADPH oxidase at Cys890, leading to its inhibition of ROS generation (Yun et al., 2011). Melatonin, which has the ability to bind NO, reduces S-nitrosation in NADPH oxidase molecules. Using *Solanum lycopersicum* as an example, it was found that treatment with exogenous melatonin first led to the accumulation of endogenous melatonin, which then removed the generated NO molecules, causing denitrosation of Cys890 residues in RBOH and increasing its activity (Gong et al., 2017) (**Figure 3**). Thus, activation of RBOH and increased ROS generation have been shown to be critical for melatonin to enhance plant resistance to drought, heat, and osmotic stress. Inhibition of RBOH or chemical removal of H_2_O_2_ by its scavenger significantly downregulated melatonin-induced plant defence responses, including decreased expression of several stress-related genes (*CDPK1*, *MAPK1*, *TSPMS*, *ERF4*, *HSP80*, and *ERD15*), and caused decreased activity of antioxidant enzymes (SOD, catalase, and ascorbate peroxidase) (Gong et al., 2017). The role of ROS generated by different molecular forms of NADPH oxidase in the stomatal closing effect induced by exogenous melatonin in *Arabidopsis* and dependent on the synthesis of NO was demonstrated (Wang et al., 2023a, see below).

Nevertheless, the mechanisms underlying the increase in NADPH oxidase activity upon melatonin treatment of plants do not seem to be limited to its effect on S-nitrosation of Cys890 residues. In particular, there is reason to believe that melatonin may activate NADPH oxidase by affecting Ca^2+^ homeostasis. For example, in watermelon plants, it was shown that the recruitment of ROS generated by NADPH oxidase and calcium ions entering the cytosol through cyclic nucleotide-gated ion channels realise the protective effect of melatonin during cold stress (Chang et al., 2021). Our experiments showed that the melatonin-induced increase in heat tolerance of wheat seedlings was abolished by the H_2_O_2_ scavenger dimethylthiourea, the NADPH oxidase inhibitor imidazole, and various calcium antagonists (Kolupaev et al., 2024b). It is noteworthy that the activation of H_2_O_2_ accumulation under the action of melatonin was not manifested in the case of seedlings treated with EGTA, a chelator of extracellular calcium, or with neomycin, an inhibitor of phospholipase C, which is involved in formation of inositol 1,4,5-phosphate and thus in opening of intracellular Ca^2+^ channels. There is reason to believe that under the above experimental conditions, melatonin-induced changes in Ca^2+^ homeostasis are primordial in relation to activation of NADPH oxidase and increased generation of ROS, which act as signalling mediators (**Figure 3**). According to one model, calcium activates a Ca^2+^-dependent protein kinase that phosphorylates the N-terminal region of the membrane-associated subunit (RBOH) of NADPH oxidase and causes its conformational change to facilitate the binding of its cytosolic component, the Rop protein (GTPase). This results in the formation of an active dimer, leading to increased ROS generation (Wong et al., 2007). In a form of the membrane-bound subunit of rice NADPH oxidase (OsRBOHB), the presence of EF arms in the N-terminal region was found, indicating the formation of a dimer involving Ca^2+^ (Oda et al., 2010). Thus, the increase in NADPH oxidase activity with the involvement of calcium may be related not only to its activation of the protein kinase, but also to the direct interaction of Ca^2+^ with the catalytic subunit. In this context, it is possible that ROS and cytosolic Ca^2+^, as mediators of melatonin effects, may functionally interact according to the principle of a mutually reinforcing “signalling loop”.

In general, several experimental data were obtained on the participation of NADPH oxidase in signalling processes necessary for the realisation of the stress-protective effect of melatonin. For example, the role of NADPH oxidase in the melatonin-induced development of tolerance to salinity has been demonstrated in *Arabidopsis*. It was found that melatonin treatment could enhance antioxidant defence in stressed wild-type plants but not in the *atrbohF* mutant (Chen et al., 2017). Exogenous melatonin treatment significantly reduced the phytotoxic effects of the pesticide chlorothalonil on tomato plants. Moreover, the melatonin-induced increase in the activity of glutathione cycle enzymes involved in chlorothalonil detoxification was abolished by treating plants with NADPH oxidase inhibitor and H_2_O_2_ scavenger. This suggests a role for ROS generated by NADPH oxidase in the realisation of the stress-protective effect of melatonin (Peng et al., 2023). The activating effect of ROS and Ca^2+^ on the enzymatic antioxidant system is thought to be mediated mainly by MAP kinase signalling cascade, with the involvement of calmodulin-dependent protein kinases (Dvořák et al., 2021) (**Figure 3**). However, there is evidence for the involvement of the MAPK signalling cascade in shaping melatonin-mediated responses to abiotic and biotic stressors (Lee and Back, 2016; 2017; Mansoor et al., 2024). For example, the expression of MPK3 and MPK6 in Arabidopsis has been shown to be induced by melatonin and 2-OHM (Lee and Back, 2016). Thus, it is conceivable that different kinases are involved in the transduction of ROS and Ca^2+^ signals activated by melatonin and are required for its induction of the antioxidant system and possibly other plant defence responses (**Figure 3**).

However, melatonin may not only have an activating effect on NADPH oxidase and ROS generation. It has been shown that melatonin can also suppress the expression of genes encoding plasma membrane-bound NADPH oxidase (*TaRbohD*, *TaRbohF*) in wheat. Such data were obtained while studying the effect of priming wheat seeds and seedlings of *Brassica juncea* with melatonin, which mitigated the subsequent toxic effects of chromium (Lei et al., 2021; Kour et al., 2024). The authors consider this effect as an additional mechanism to mitigate oxidative stress. Similar effects were found in a study on the protective effects of melatonin on *Vicia faba* plants exposed to arsenic toxicity (Siddiqui et al., 2020). Melatonin treatment reduced the effects of increased NADPH oxidase activity and O_2_^•−^ and H_2_O_2_ generation induced by the treatment. At the same time, no inhibitory effect of melatonin on NADPH oxidase activity and ROS generation was observed in the absence of stress stimuli. It is still unclear how the effects of NADPH oxidase activation and suppression by melatonin recorded in different objects relate to each other. A different set of mediators may be involved in the regulation of NADPH oxidase under stress conditions than under optimal conditions. Moreover, the regulation of redox homeostasis during the action of exogenous melatonin on plants may be even more complex because of its transformation into hydroxy derivatives. For example, Lee and Back (2021) obtained experimental data suggesting a more important role of 2-OHM in enhancing ROS generation than melatonin. It was shown that treatment of tobacco and *Arabidopsis* leaves with 2-OHM resulted in a significantly greater enhancement of superoxide anion radical generation compared to melatonin treatment.

Recently, more significant effects of 2-OHM on *Arabidopsis* seed germination than melatonin have been reported (Lee and Back, 2022). These effects are attributed to ROS-mediated induction of gibberellin synthesis. Molecular genetic studies using the knockout mutant *m2h* and plants with overexpression of the M2H gene confirmed the importance of increasing ROS generation in the implementation of the effect of 2-OHM on the expression of the gibberellin synthesis enzyme gene and seed germination (Lee and Back, 2022). Undoubtedly, data on the participation of 2-OHM and other metabolites of melatonin in cellular signalling processes are still insufficient. It is still very difficult to distinguish the physiological effects of these compounds from those of melatonin itself.

## Functional relationships between melatonin and NO

GT NO, together with ROS, is considered a very important signalling mediator of plant cells, somehow related to almost all known signalling molecules (Santolini et al., 2017). One of the main ways of realising the signalling potential of NO molecules is through the PTM of proteins. The most common NO-mediated PTM is S-nitrosation, a reversible redox modification based on the addition of a nitroso group to the thiol group (SH) of a specific cysteine residue (Cys) to form S-nitrosothiol (SNO), which can induce conformational changes and, consequently, alter the activity or function of the corresponding protein (Lamotte et al., 2015). To date, hundreds of proteins that are regulated by S-nitrosation have been identified (Sánchez-Vicente et al., 2019). The second most abundant NO-mediated PTM is tyrosine nitration (Blume et al., 2013; Sánchez-Vicente et al., 2019; Zhu et al., 2019).

The biological significance of this process is still difficult to interpret, as it is considered one of the markers of nitrosative stress (Ischiropoulos, 2003). It is likely that tyrosine nitration is also important in plants for regulating the activity of individual proteins. For example, nitration of tyrosine residues in molecules of the major microtubule protein α-tubulin has been found, which may be related to the regulation of their dynamic properties and participation in the growth and division of plant cells (Blume et al., 2013).

NO is considered to be one of the main regulators of Ca^2+^ homeostasis. It affects several types of Ca^2+^ channels and promotes Ca^2+^ entry into the cytosol from the extracellular space and intracellular compartments (Jeandroz et al., 2013; Santolini et al., 2017). NO, through S-nitrosation, affects the functional activity of many proteins involved in Ca^2+^ signalling, including protein kinases (Courtois et al., 2008) and calmodulin (Astier et al., 2012; Jeandroz et al., 2013). It is also evident that the targets of NO-mediated PTM are also proteins involved in Ca^2+^ channel opening. Studies using agonists or antagonists of cGMP and cADP-ribose (cADPR) have shown that these secondary cytosolic messengers play a central role in the realisation of the effects of NO on Ca^2+^ channels (Santolini et al., 2017). There is also considerable experimental evidence for NO activation of the mitogen-activated protein kinase (MAPK) cascade, which in turn is tightly coupled to many other components of the signalling network (Zhu et al., 2019).

The signalling potential of NO is also realised by its functional and chemical interaction with ROS, in particular with O_2_^•−^ and H_2_O_2_. This interaction of key signalling mediators can be either synergistic or antagonistic (Farnese et al., 2016). NO-induced PTMs of individual protein molecules can induce rapid and long-lasting effects that may have different directions. For example, inhibition of individual antioxidant enzymes by the direct action of NO may lead to the formation of a signal that induces gene expression of these enzymes (Corpas et al., 2022b; Kolupaev et al., 2023b). On the other hand, NO-induced PTMs can reduce the activity of enzymes involved in ROS generation, such as glycolate oxidase and NADPH oxidase, allowing the cell to maintain redox homeostasis (Farnese et al., 2016). To date, there is evidence that NO is also involved in the realisation of the physiological effects of most known plant hormones (Zhou et al., 2019; Shang et al., 2022; Kolupaev et al., 2024a). Melatonin is a new plant hormone, but there is already considerable evidence of its functional and chemical interactions with NO (He and He, 2020). This interaction is manifested by melatonin binding NO to form nitrosomelatonin, influencing S-nitrosation processes, and inducing NO synthesis (Corpas et al., 2022a; Ahmad et al., 2023).

### Pathways of nitric oxide synthesis in plants

It is now generally accepted that there are two main pathways for the synthesis of NO in plants: a reductive pathway from nitrates and nitrites, which is mainly driven by nitrate reductase (NR) activity, and an oxidative pathway associated with the conversion of L-arginine by an enzyme with activity similar to NO synthase (NOS), so called because its activity requires the same biochemical conditions as animal NOS (Astier et al., 2018; Corpas et al., 2022a). In higher plants, the main cellular compartments of NO generation are the cytosol, peroxisomes, chloroplasts, and mitochondria. Experimental data indicate that NO can be synthesized in the cytosol with the participation of NR, a multifunctional enzyme involved in nitrogen assimilation and metabolism. *In vivo* and *in vitro* experiments have shown that NR can catalyse the reduction of nitrate to NO and its derivative peroxynitrite (ONOO^−^) (Khator et al., 2024). The activity of different molecular forms of NR can be regulated by phosphorylation involving MAP kinases, whose activity can be modulated by ROS (Wang et al., 2010; Zhu et al., 2019).

NO can be synthesized in mitochondrial membranes *via* the reductive pathway, but with the participation of other catalytic systems. NR as well as the enzymatic complexes of the electron transport chain cytochrome oxidase (CIII) and cytochrome reductase (CIV) can be involved in this process (Gupta and Kaiser, 2010; Farnese et al., 2016; Khator et al., 2024). The oxidative pathway of NO synthesis is considered to be as important as the reductive pathway, although the nature of the enzymatic systems that provide this pathway in higher plants has been debated for nearly three decades (Farnese et al., 2016). Nevertheless, enzymatic oxidation of L-arginine to citrulline and NO has been shown to be possible in leaf peroxisomes and chloroplasts of green algae and vascular plants (Hancock and Neill, 2019). This enzymatic activity has been named NOS-like because, as in the case of the animal enzyme, it has been reported to be strictly dependent on the presence of arginine and NADPH as well as several NOS cofactors (NADPH, FAD, FMN, Ca^2+^, and calmodulin) (Corpas and Barroso, 2014; Farnese et al., 2016). However, molecular genetic evidence of the presence of the corresponding protein in higher plants is still lacking (Astier et al., 2018). Currently, there is a hypothesis that there are polypeptides with redox-active domains that can be assembled into a single enzymatic complex that catalyses the reactions of arginine-dependent NO formation in higher plants (Kolbert et al., 2019). Partial overlap of cellular compartments where NO is synthesised with those for melatonin synthesis (chloroplasts and mitochondria) is considered by some authors as a fact that indirectly indicates a possible relationship between melatonin and NO (Wang et al., 2022a).

### Nitric oxide conjugates and their functional relationship to melatonin

A peculiarity of NO is its short half-life (approximately 30 s) (Martínez-Lorente et al., 2022). In this respect, its physiological effects depend largely on its transformation into more stable conjugated compounds. Among these, S-nitrosothiols are well known. The most abundant S-nitrosothiol is S-nitrosoglutathione (GSNO), which is formed by the interaction of NO with reduced glutathione (GSH) (Corpas et al., 2013). GSNO levels are regulated by S-nitrosoglutathione reductase, which reduces GSNO to glutathione sulfinamide (GS(O)NH_2_) using NADH (Martínez-Lorente et al., 2022). Melatonin may affect the GSNO pool by inhibiting S-nitrosoglutathione reductase (Wen et al., 2016).

An even more important mechanism of interaction between NO and melatonin may be the formation of NOMela, which occurs in an aerobic environment and at physiological pH values (Martínez-Lorente et al., 2022). This interaction is considered one of the mechanisms of NO binding to prevent the development of nitrosative stress (Singh et al., 2016). At the same time, NOMela is considered one of the natural NO donors, like GSNO or S-nitrosocysteine (Mukherjee, 2019). Both nitrosothiols and NOMela can participate in protein PTM as NO sources. The ability of NOMela to efficiently transnitrosate cysteine residues in proteins has been demonstrated using an *in vitro* system (Singh et al., 2016). In particular, NOMela was found to be 10 times more effective than S-nitrocysteine in the reaction of S-nitrosation of catalytically active cysteine residues by glyceraldehyde-3-phosphate dehydrogenase (Kirsch and de Groot, 2008).

NOMela may be a conjugate that enables long-distance transport of both melatonin and NO at the whole-plant level (Mukherjee, 2019). It has been suggested that GSNO and NOMela may compete for binding sites for long-distance transport in plants, although direct experimental evidence for this hypothesis is still lacking (Singh et al., 2016). However, NOMela has been shown to be more efficient than GSNO in transporting NO from roots to leaves of *Arabidopsis* in a model experiment (Singh et al., 2021).

### Effect of melatonin on NO synthesis in the regulation of plant resistance to stress

**Figure 4:**
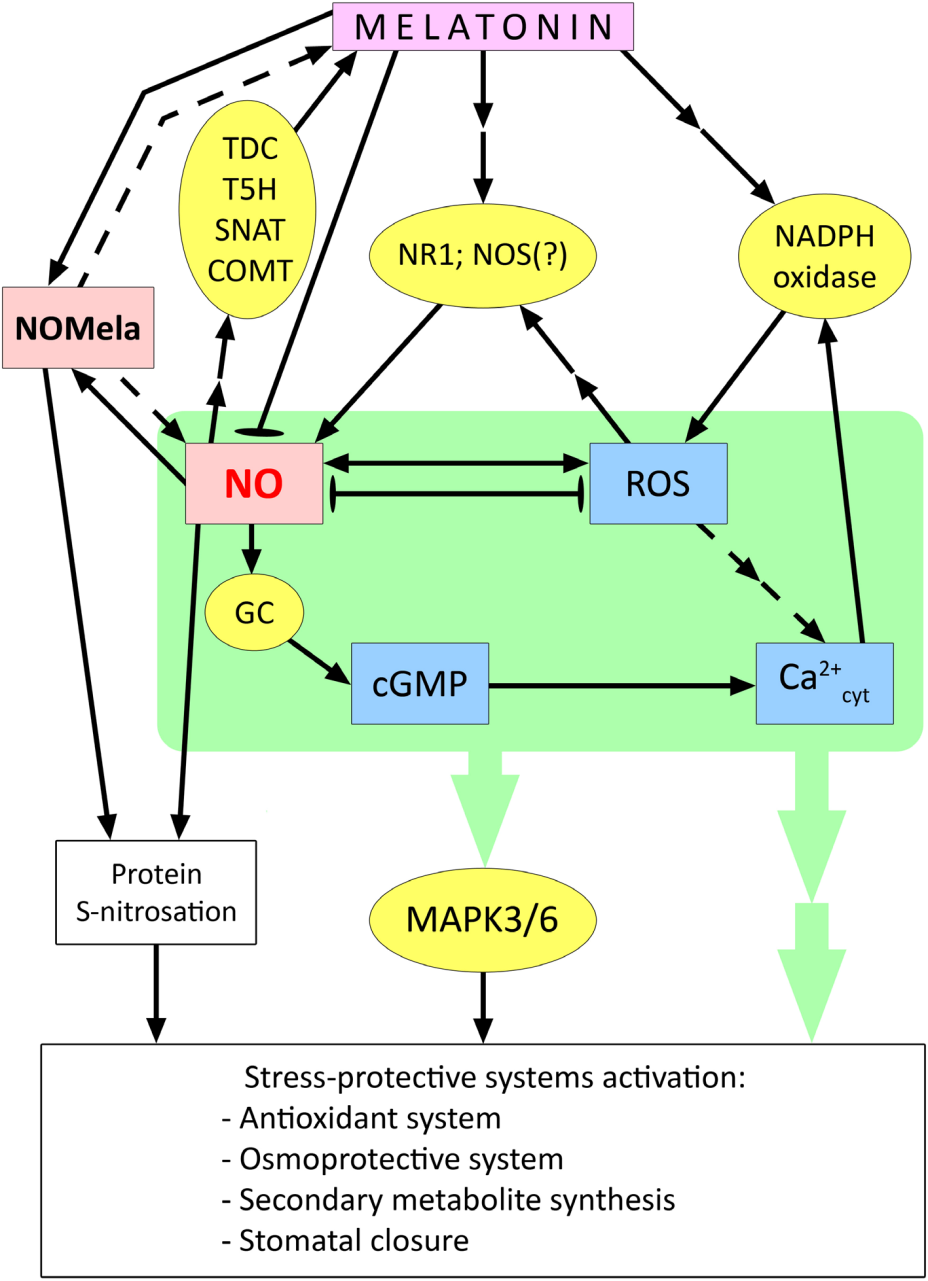
Involvement of NO in the activation of stress protection systems in plant cells under the action of melatonin. cGMP – cyclic guanosine monophosphate; COMT – caffeic acid O-methyltransferase; GC – guanylate cyclase; MAPK3/6 – mitogen activated protein kinases 3/6; NOMela – N-nitrosomelatonin; NOS – NO synthase; NR1 – nitrate reductase 1; ROS – reactive oxygen species; SNAT – serotonin N-acetyltransferase; T5H – tryptamine 5-hydroxylase; TDC – tryptophan decarboxylase. Dashed arrows indicate connections between signalling mediators without clear experimental evidence; blunt-ended arrows indicate antagonistic interactions between signalling mediators. Other explanations in the text.

Several studies have reported an increase in NO synthesis during melatonin treatment of plants and the role of this effect in the induction of plant resistance to various stress stimuli (**Table 1**; **Figure 4**). For example, treatment of tomato plants with melatonin, which induces the development of heat tolerance, resulted in an NR-dependent increase in leaf NO content (Jahan et al., 2019). Data on the participation of NO as a mediator of the action of melatonin were also obtained when studying the induction of cold resistance in plants, in particular rape seedlings (Ma et al., 2022). The increase in NO and cytosolic Ca^2+^ was more significant in melatonin-treated leaves in response to cold than in untreated leaves (**Table 1**). Notably, co-treatment of melatonin-treated plants with NO or Ca^2+^ antagonists also inhibited melatonin-induced MAPK3/6 expression under cold stress conditions (Ma et al., 2022). Thus, there is reason to believe that Ca^2+^ and NO are at the beginning of the melatonin-induced signalling cascade leading to the development of cold tolerance in rapeseed.

**Table 1:** Examples of the involvement of nitric oxide in the realisation of the effect of melatonin on plant resistance to abiotic stresses.

Stressor	Plant species	Physiological effects of melatonin	Experimental evidence of NO involvement in melatonin effects	Source
High temperatures	*Solanum lycopersicum* L.	Increased heat resistance	Increased NO levels in leaves due to increased NR activity and enhanced *NR* gene expression	Jahan et al., 2019
Low temperatures	*Brassica napus* L.	Increased resistance to low temperatures	Increase in cytosolic Ca^2+^, NO content in leaves with subsequent increase in MAPK3/6 expression. Elimination of melatonin effects in the background of NOS (L-NAME) and NR (tungstate) inhibitors and Ca^2+^ channel blocker LaCl_3_ or Ca^2+^ chelator EGTA	Ma et al., 2022
	*S. lycopersicum*	Improvement of fruit storage at low temperatures	Enhanced *NOS1* gene expression and increased NOS-like activity	Aghdam et al., 2019
Salinisation	*B. napus*	Mitigation of growth inhibition, reduction of oxidative damage, preservation of ionic homeostasis	Increased NO levels in seedlings, increased levels of S-nitrosated proteins. Elimination of stress protective effect of melatonin by NO scavenger PTIO	Zhao et al., 2018
	*Glycine max* L.	Reduction of growth inhibition, enhancement of isoflavone accumulation	Increase in NO levels in seedlings, accompanied by increased expression of *NR1*, *NR2* and *NOA1* genes. Elimination of melatonin effects on plant growth and secondary metabolite accumulation in the presence of cPTIO	Yin et al., 2022
UV-B	*G. max*	Reduction of growth inhibition, activation of antioxidant system, accumulation of isoflavones, enhancement of gene expression of enzymes involved in their synthesis	Increased NR activity and enhanced *NR1* and *NR2* gene expression. Elimination by cPTIO treatment of the effects of increased gene expression of isoflavone synthesis enzymes and accumulation of these secondary metabolites.	Yin et al., 2023
Cd toxicity	*Triticum aestivum* L.	Mitigation of oxidative stress manifestations	Enhancement of NO synthesis, elimination of stress-protective effect of melatonin by plant PTIO treatment	Kaya et al., 2019
	*T. turgidum* L.	Reduction of seedling growth inhibition, mitigation of oxidative damage	Increase in endogenous NO levels and NR and nitrite reductase activities	Aloui et al., 2024
	*Catharanthus roseus* L.	Mitigation of oxidative damage, increased proline synthesis, increased activity of antioxidant enzymes	Elimination of melatonin stress-protective effect by cPTIO	Nabaei and Amooaghaie, 2019

In addition, the involvement of NO in the preservation of melatonin-treated tomato fruits during storage at low temperatures was demonstrated (Aghdam et al., 2019).

One of the effects of exogenous melatonin, which is important for plant resistance to drought and other stresses associated with plant desiccation, is stomatal closure. These processes depend on NO and its functional interaction with ROS. In *Arabidopsis* plants, it was shown that exogenous melatonin-induced stomatal closure was reversed by the NO scavenger cPTIO (carboxy-phenyl-4,4,5,5-tetramethylimidazoline-1-oxyl-3-oxide) (Wang et al., 2023a). In addition, lines mutant for the genes for NO synthesis enzymes, nitrate reductase 1 and 2 (*nia1/nia2*) and NO-associated 1 (*noa1*), were found to be unable to close stomata when exposed to melatonin. Melatonin-induced NO production was also impaired in plants mutant for NADPH oxidase genes (*rhohC* and *rbohD/F*). This indicates that NO is downstream of ROS in the melatonin-inducible signalling chain (Wang et al., 2023a) (**Figure 4**).

NO may also mediate the induction of salt tolerance by exogenous melatonin in rapeseed (**Table 1**). In addition, it was shown that mutants defective in NR genes had increased sensitivity to salt stress and that melatonin did not affect their salt tolerance (Zhao et al., 2018).

Using soybeans as an example, it has been shown that when melatonin induces salt tolerance and UV-B resistance NO is involved in an important defence response such as the accumulation of isoflavones (Yin et al., 2022, 2023) (**Table 1**).

The involvement of NO was also shown in the protective effect of melatonin on plants of two wheat species and *Catharanthus roseus* exposed to cadmium toxicity (**Table 1**). Notably, treatment of *C. roseus* plants with an NO donor, as with melatonin, increased proline content and antioxidant enzyme activities in roots under Cd stress (Nabaei and Amooaghaie, 2019). These melatonin-induced responses were inhibited by the NO scavenger, cPTIO. However, different results were obtained when the effect of melatonin on the Cd resistance in Chinese cabbage (*Brassica rapa* subsp. *pekinensis*) was studied (Wang et al., 2021). It was shown that treatment of the plants with melatonin significantly reduced the Cd content in them. At the same time, treatment with the NO scavenger cPTIO had the same effect on the Cd content in the plants. The authors showed that NO enhances the expression of the Cd transporter gene IRT1, thereby increasing Cd uptake and aggravating stress in plants, while exogenously added melatonin can inhibit NO synthesis, thereby reducing Cd levels and alleviating stress caused by its toxic effect (Wang et al., 2021).

The effect of reducing NO levels in plants treated with melatonin has also been reported in some other studies. There are reasons to believe that melatonin, in addition to inducing the synthesis of NO as a signalling mediator necessary for the activation of stress-protective systems, can prevent the accumulation of excessive amounts of NO and the development of nitrosative stress in plants.

Apparently, there are complex direct and inverse relationships between melatonin and NO. In addition to the facts of melatonin modulation of NO synthesis discussed above, data have also been obtained on the ability of NO to act as a signal inducing melatonin synthesis. Thus, it has been shown that NO mediated by cGMP can activate the expression of genes for the melatonin synthesis enzymes TDC, T5H, SNAT, and COMT, which leads to an increase in the endogenous level of melatonin (Wang et al., 2022a; Aghdam and Arnao, 2024). The NO scavenger cPTIO was found to disrupt Cd-induced melatonin synthesis by decreasing the expression of TDC and COMT genes in rice (He and He, 2020). Thus, plants may possess mechanisms for the mutual enhancement of melatonin and NO synthesis. At the same time, melatonin, which is synthesized in chloroplasts and mitochondria, may directly reduce the toxic effect of RNS in these organelles (Wang et al., 2022a).

Summarising the available data, it can be assumed that cytosolic Ca^2+^- and ROS-dependent enhancement of NO formation is a tool for melatonin activation of other components of the signalling network (in particular, the MAP-kinase cascade) and subsequent changes in the expression of genes that control rather universal stress-protective systems (antioxidant complex, synthesis of osmoprotectants, secondary metabolites, stomatal closure, etc (He and He, 2020; Khan et al., 2023; Plokhovska et al., 2023; Aghdam and Arnao, 2024) (**Figure 4**). On the other hand, the functional interaction between melatonin and NO also includes mechanisms that limit the effects of NO, in particular the formation of NOMela conjugate, as well as the negative effect of melatonin on the gene expression of NO synthesis enzymes, the mechanisms of which remain unclear. Thus, melatonin may prevent the development of nitrosative stress in plants. At the same time, it remains unclear how the transition from a synergistic interaction between melatonin and NO to an antagonistic one can occur. The complexity of the functional interaction between melatonin and NO is further complicated by the ambiguous and not yet well-understood role of NOMela, which may be both a product of excess NO binding and a source of NO for S-nitrosation processes of some proteins (**Figure 4**).

## Functional relationships of melatonin and hydrogen sulfide in plants

Hydrogen sulfide (H_2_S) in animal cells is considered the third GT in terms of time of discovery and the importance after NO and CO (Liu et al., 2024). However, in the context of plant cell function, evidence for the role of H_2_S and related signalling processes is accumulating more dynamically than for the action of CO. Numerous studies have shown that H_2_S acts as a gaseous signalling molecule that enhances plant adaptation/tolerance to various abiotic stresses, including drought, salinity, temperature extremes, heavy metal effects, and other adverse factors (Zhang et al., 2021; Liu et al., 2024). H_2_S has also been found to be directly or indirectly involved in a wide range of physiological processes that occur under normal conditions, including seed germination, root development, stomatal movement, and fruit ripening (Corpas and Palma, 2020). At the same time, H_2_S is closely related to other components of the signalling network, primarily ROS, calcium ions, and NO (Corpas et al., 2022b; Khan et al., 2022; Liu et al., 2024). One of the most studied ways in which H_2_S is involved in signalling processes is through a PTM of proteins known as persulfidation (Liu et al., 2024). This process involves the conversion of protein thiol groups (RSH) to persulfide groups (RSSH). It is suggested that such a reversible PTM is not only important for signalling processes but is also one of the mechanisms of the direct protective effect of H_2_S on proteins under conditions of oxidative stress (Wang et al., 2021). The mechanism of protein persulfidation is not fully understood. It is believed that H_2_S or its ionic forms, HS^–^ and S_2_^–^, cannot react directly with protein thiols. Such interaction requires the presence of oxidizing agents (Aroca et al., 2021).

Persulfidation is likely a part of the toolkit for gene expression regulation. A transcriptome study performed on *Arabidopsis* plants showed that treatment with exogenous H_2_S induced significant changes in the expression of genes encoding different transcriptional regulators (Aroca et al., 2017). Persulfidation is also one of the mechanisms regulating the activity of several antioxidant enzymes, and it can cause both increased and decreased catalytic activity and protect protein molecules from oxidative degradation (Li et al., 2020; Shivaraj et al., 2020). In addition, the same enzymatic proteins can be targets for persulfidation and S-nitrosation, which is a prerequisite for functional interaction between H_2_S and NO (Kolupaev et al., 2023b). For example, the possibility of cross-regulation of activity by persulfidation and nitrosation has been demonstrated for several molecular forms of ascorbate peroxidase (Kolupaev et al., 2023b; Li et al., 2024). It was also shown that persulfidation of two cysteine residues increased the activity of one of the molecular forms of NADPH oxidase (Shen et al., 2020), suggesting a functional interaction between H_2_S and ROS in signalling processes. On the other hand, as mentioned above, NO can inhibit NADPH oxidase through S-nitrosylation. Thus, ROS, NO, and H_2_S form a complex signalling and regulatory network that controls redox modifications of proteins (Kolupaev et al., 2023b).

Another integral component of such a network is calcium as a universal intracellular messenger. In particular, the possibility of persulfidation of Ca^2+^ channels with an increase in the concentration of H_2_S in the submembrane space is considered (Li et al., 2024). In many works, the dependence of induction of physiological reactions by exogenous H_2_S (its donors) on Ca^2+^ homeostasis has been shown by inhibitor methods (Li et al., 2012; Kolupaev et al., 2017).

### Brief overview of H_2_S synthesis in plants

In plant cells, H_2_S synthesis occurs predominantly in chloroplasts and to a lesser extent in other subcellular spaces such as the cytoplasm and mitochondria (Pandey et al., 2024). L-cysteine desulfhydrase (LCD) is considered to be the major enzyme of H_2_S synthesis. It is localised in cytoplasm, plastids and mitochondria as reported by (Riemenschneider et al., 2005). The synthesis of H_2_S from D-cysteine is also possible through the action of D-cysteine desulfhydrase localised in the cytoplasm (Guo et al., 2016). In recent years, the desulfhydrase AtDES1 has been considered another novel H_2_S synthesising enzyme in the *Arabidopsis* cytoplasm, which is believed to be a protein similar to cysteine synthase, but mainly exhibits activity related to the degradation of L-cysteine, accompanied by the formation of H_2_S (Zhang et al., 2021). Experimental evidence has been provided for the role of this enzyme in the synthesis of H_2_S in response to abiotic stresses, particularly drought (Zhang et al., 2021).

In chloroplasts, H_2_S can also be synthesized by sulfite reduction involving sulfite reductase when the photosynthetic sulfate assimilation pathway is active (Pandey et al., 2024). In mitochondria, H_2_S formation is possible during cyanide detoxification. This process is mediated by β-cyanoalanine synthase, an enzyme that catalyses the conversion of cyanide to β-cyanoalanine with the consumption of cysteine (Feng et al., 2022). The putative compartmentalisation of the synthesis of a significant pool of H_2_S in mitochondria and chloroplasts, compartments in which melatonin is mainly synthesised, is considered the basis for studying the functional interaction between melatonin and H_2_S (Wang et al., 2022b).

### Involvement of H_2_S in realisation of stress-protective effect of melatonin on plants

At present, there is a lot of data indicating the participation of H_2_S as a mediator in the realisation of the protective effects of melatonin when plants are exposed to stress factors of various nature (**Table 2**). In particular, it was shown that the increase in heat resistance of wheat plants under the influence of melatonin was eliminated by the H_2_S scavenger hypotaurine (Iqbal et al., 2021). The dependence of melatonin enhancement for drought tolerance in tomato plants on H_2_S synthesis has also been reported (Khan et al., 2024a) (**Table 2**).

**Table 2:** Examples of the involvement of hydrogen sulfide in the realisation of the effect of melatonin on plant resistance to abiotic stresses.

Stressor	Plant species	Physiological effects of melatonin	Experimental evidence of H_2_S involvement in melatonin effects	Source
High temperatures	*Triticum aestivum* L.	Increased plant heat tolerance, photosynthetic activity and carbohydrate content	Elimination of melatonin effects by H_2_S scavenger hypotaurine	Iqbal et al., 2021
Drought	*Solanum lycopersicum* L.	Increased drought tolerance with increased activity of mitochondrial enzymes and increased synthesis of stress proteins HSP17.6 and HSP70	Elimination of melatonin effects by H_2_S synthesis inhibitor propargylglycine	Khan et al., 2024a
	*Arabidopsis thaliana* L.	Activation of drought defence systems: increased expression of genes for the transcription factors CBF2, CBF3, RD29A, DREB2A and DREB2B, as well as genes related to K^+^ channels of stomatal closing cells	Increase in endogenous H_2_S levels and increase in expression of genes for H_2_S synthesising enzymes, *LCD* and *DES1*. Almost complete absence of melatonin stress protective effect in *lcd*, *des1*, and *lcd/des1* mutants	Wang et al., 2023b
Salinisation	*S. lycopersicum*	Mitigation of stress-induced growth inhibition and membrane damage	Increase in activity and appearance of new L-DES isoforms, increase in H_2_S content	Mukherjee and Bhatla, 2021
		Maintenance of K^+^/Na^+^ homeostasis in seedling roots, increase in antioxidant enzyme activity, mitigation of oxidative damage	Increase in L-DES activity and H_2_S content. Elimination of melatonin protective effects by H_2_S scavenger hypotaurine	Siddiqui et al., 2021
	*Satureja hortensis* L.	Mitigation of growth inhibition, maintenance of K^+^/Na^+^ homeostasis, protection of photosynthetic apparatus from toxic effects of ions, enhancement of essential oil biosynthesis	Elimination of melatonin protective effects by H_2_S scavenger hypotaurine and their enhancement by H_2_S donor	Khalofah et al., 2024
	*T. aestivum*	Mitigation of salinity stress by increased activity of antioxidant system and upregulated expression of Na^+^ transport genes (*SOS1*, *SOS2*, *SOS3*, *NHX1*), improved photosynthetic parameters	Elimination of melatonin effects by H_2_S synthesis inhibitor propargylglycine	Khan et al., 2024c
Combined effects of drought and salinity	*S. lycopersicum*	Activation of enzymatic and non-enzymatic antioxidant systems, accumulation of osmolytes, maintenance of ion homeostasis	Elimination of melatonin protective effects by H_2_S scavenger hypotaurine, synergistic effect of melatonin and H_2_S donor on functioning of stress-protective systems	Khan et al., 2024b
As toxicity	*Capsicum annuum* L.	Mitigation of As-induced growth inhibition and oxidative stress, As localisation in vacuoles	Increased H_2_S content, reduced protective effect of melatonin by H_2_S scavenger hypotaurine and its enhancement by H_2_S donor NaHS	Kaya et al., 2022
	*S. lycopersicum*	Reduction of As uptake, activation of the synthesis of phenolic and flavonoid compounds and phytochelatins	Inhibition of melatonin effects by hypotaurine and their enhancement by H_2_S donor NaHS	Ghorbani et al., 2024
Cr toxicity	*S. lycopersicum*	Enhancement of phytochelatin synthesis, increase in H^+^-ATPase activity, stabilisation of ionic homeostasis	Inhibition of melatonin effects by H_2_S scavenger hypotaurine	Khan et al., 2023
Pb toxicity	*Carthamus tinctorius* L.	Decrease in stress-induced lipoxygenase activation, decrease in ROS and lipid peroxidation products; increase in antioxidant enzyme activity and increase in phytochelatins synthesis	Enhancement of melatonin effects by H_2_S donor NaHS	Haghi et al., 2022

In *Arabidopsis* plants, molecular genetic methods have shown that H_2_S is involved in many of the stress-protective effects of melatonin under drought (Wang et al., 2023b) (**Table 2**). It was also found that both melatonin and H_2_S affected the level of transcription of genes associated with K^+^ channels involved in maintaining stomatal closure (Wang et al., 2023b).

**Figure 5:**
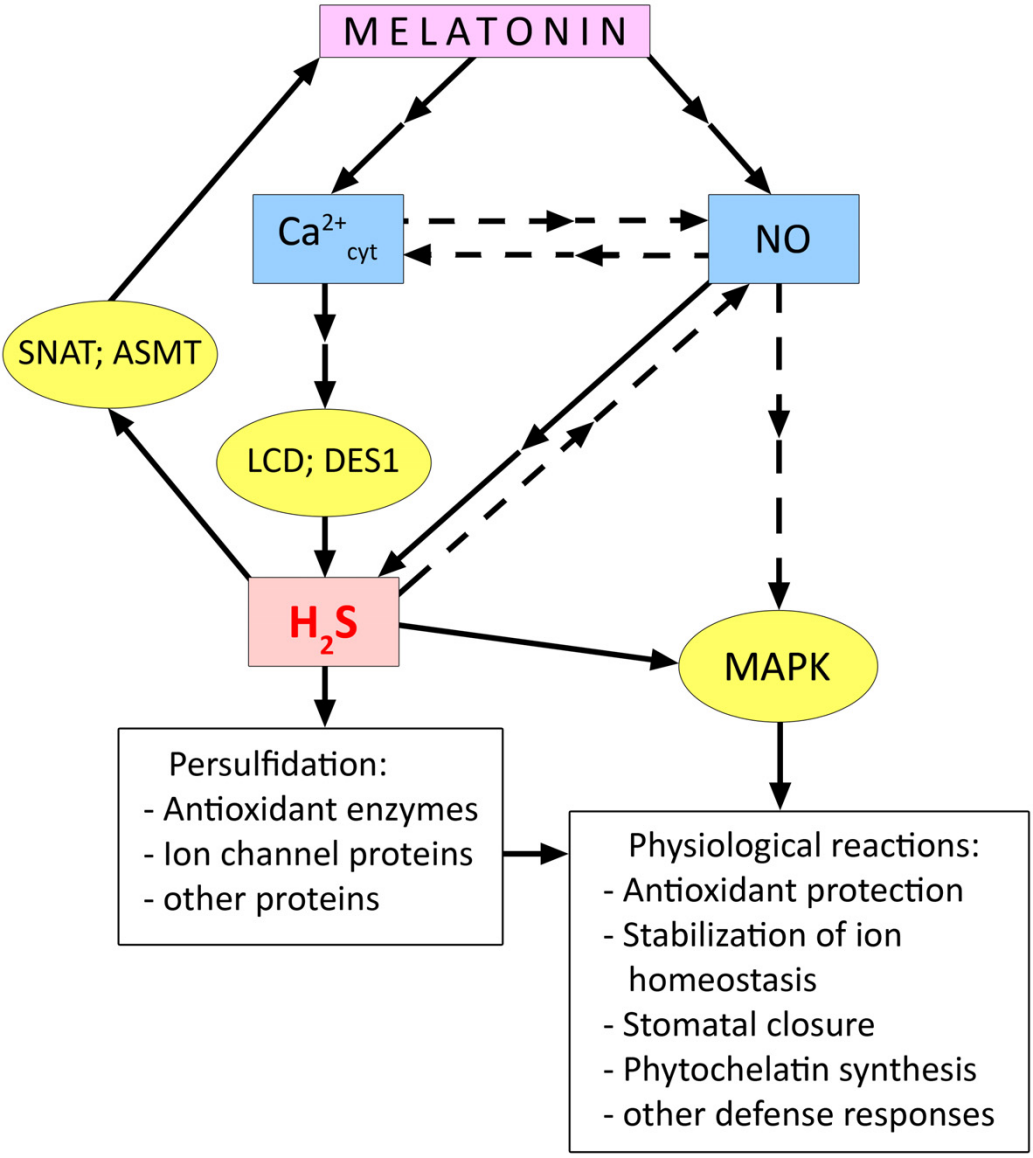
Relationship between melatonin and hydrogen sulfide during activation of plant cell stress defence systems. ASMT, N-acetylserotonin O-methyltransferase; DES1, cysteine synthase-like desulfhydrase 1; LCD, L-cysteine desulfhydrase; MAPK, mitogen activated protein kinases; SNAT, serotonin N-acetyltransferase. Dashed arrows indicate connections between signalling mediators without clear experimental evidence. Other explanations in the text.

There is reason to believe that not only the effect of melatonin on stomatal status depends on H_2_S synthesis, but conversely, H_2_S-induced stomatal closure depends on melatonin synthesis (Wang et al., 2022a). For example, *Arabidopsis* plants showed that exogenous H_2_S increased transcript levels of the melatonin synthesis enzymes SNAT, ASMT, and COMT1 in leaves and caused persulfidation of SNAT and ASMT. At the same time, H_2_S was found to have little effect on the condition of stomata in mutants of the melatonin synthesis enzymes *snat*, *comt1*, and *asmt* (Wang et al., 2022a). These findings suggest that there may be some kind of signal amplifying loop between melatonin and H_2_S (**Figure 5**).

This hypothesis is also supported by data on the synergistic interaction between melatonin and H_2_S, obtained in the study of plant adaptation to various stresses. Thus, the involvement of endogenous H_2_S and the LCD enzyme was found in the melatonin-induced increase in salt tolerance of tomato (**Table 2**). Synergism of the joint protective effect of melatonin and H_2_S was also observed, which was expressed in the prevention of the development of oxidative cell damage by increasing the activity of antioxidant enzymes (Siddiqui et al., 2021). Data indicating the involvement of H_2_S as a mediator in the realisation of stress-protective effects of melatonin were also obtained when studying its effect on salt tolerance of *Satureja hortensis* L. (**Table 2**).

The protective effect of melatonin on the state of antioxidant and osmoprotective systems of tomato plants under the combined action of drought and salinity was not manifested in the presence of the H_2_S scavenger hypotaurine but was enhanced by simultaneous treatment of plants with a hydrogen sulfide donor (Khalofah et al., 2024; **Table 2**).

H_2_S also appears to be involved in realising the protective effects of melatonin on pepper and tomato plants exposed to arsenic toxicity (**Table 2**). These studies (Kaya et al., 2022; Ghorbani et al., 2024) also found not only attenuation of the stress-protective effect of melatonin by the H_2_S scavenger, but also enhancement of the protective effects when melatonin was used together with the H_2_S donor NaHS.

Data were obtained on the participation of H_2_S as a mediator in the development of specific protective reactions of plants induced by melatonin and in adaptation to the action of other heavy metals (**Table 2**). Thus, the melatonin-induced increase in the resistance of tomato seedlings was attenuated by an H_2_S scavenger (Khan et al., 2023). Treatment of safflower plants with melatonin and the H_2_S donor NaHS attenuated the toxic effect of lead (Haghi et al., 2022). The combined action of melatonin and NaHS was particularly effective (**Table 2**), with a significant increase in the activity of enzymes responsible for the ascorbate-glutathione cycle and increased synthesis of metal-binding ligands.

It is still unclear how H_2_S as a participant of signal transduction induced by melatonin is related to other key signal mediators. At the same time, some experimental data suggest the role of interaction of H_2_S with Ca^2+^ and NO in the manifestation of melatonin-induced stress-protective responses. Mukherjee et al. (2023) showed that enhancement of synthesis of H_2_S required for melatonin to strengthen sunflower resistance to salt was suppressed in the presence of the Ca^2+^ chelator EGTA and the Ca^2+^ channel blocker verapamil. The involvement of Ca^2+^ signalling in the activation of H_2_S synthesis was also confirmed in the study of melatonin activation of seed germination in plants of different species (Wang et al., 2024).

Exogenous melatonin caused an increase in salt tolerance of cucumber seedlings, improved the function of the photosynthetic apparatus and antioxidant system, and increased MAPK activity under stress conditions (Sun et al., 2021). All the above-mentioned stress-protective effects of melatonin were eliminated by treating plants with NO scavenger (cPTIO) and H_2_S scavenger (hypotaurine). In contrast, the MAPK inhibitor (U0126) had no effect on H_2_S and NO synthesis. The authors suggest that H_2_S acts as a mediator of the physiological effects of melatonin and is upstream of the MAPK cascade in the signalling chain (**Figure 5**). Kaya et al. (2020) provided evidence that the melatonin-induced increase in H_2_S synthesis in pepper (*Capsicum annuum* L.) plants was mediated by an increase in NO levels. The authors found that the melatonin-induced increase in H_2_S content was suppressed by the action of both the H_2_S scavenger hypotaurine and the NO scavenger cPTIO. In contrast, hypotaurine did not eliminate the effect of increased NO in plants. This work also showed the involvement of NO and H_2_S in the realisation of the protective effect of foliar treatment of pepper plants with melatonin under iron deficiency and salinity stress (Kaya et al., 2020).

Thus, few experimental data indicate the joint involvement of NO and H_2_S in transducing melatonin-induced signals leading to the development of plant resistance to certain stresses such as salinity (Kaya et al., 2020; Sun et al., 2021) (**Figure 5**). At the same time, it has been shown that treatment of etiolated seedlings of *Triticum turgidum* L. with melatonin, which increases their resistance to cadmium, was accompanied by an increase in NO levels but a decrease in H_2_S levels and the activity of its synthetic enzymes L- and D-cysteine desulfhydrases (Aloui et al., 2024). It is difficult to interpret such data on the antagonistic interaction between melatonin and H_2_S because they are sporadic and may be due to specific features of the object of study or the experimental design. In this regard, it should be noted that a recent work by Zulfiqar et al. (2024) reported a synergistic effect of melatonin and the H_2_S donor NaHS on the resistance of *Matthiola incana* L. plants to the toxic effect of cadmium. When used together, melatonin and H_2_S donor more effectively protected plants from the development of oxidative stress by increasing the activity of antioxidant enzymes and one of the key enzymes of the synthesis of secondary metabolites phenylalanine ammonia-lyase.

## Possible relationship of melatonin and carbon monoxide in plant adaptation to abiotic stresses

The mechanisms underlying the biological activity of CO are significantly different from those of NO and H_2_S. It is assumed that they are largely due to the formation of coordination bonds between CO and metals in the active centres of proteins, primarily heme-containing proteins (Feelisch and Olson, 2013). In general, however, the question of the molecular targets of CO action involved in the manifestation of certain physiological effects in plants remains open. Nevertheless, it is now clear that CO has a positive effect on seed germination, root development, fruit ripening and maturation, and stomatal closure (Cao et al., 2007; Zhang et al., 2014; Gahir et al., 2020; Hong et al., 2024). In recent years, the involvement of CO in the adaptation of plants to the action of stress factors of different nature and the possibility of increasing plant resistance with the help of CO donors have been studied particularly intensively (Yao et al., 2019; Kolupaev et al., 2022).

Hemoxygenase, which catalyses the stereospecific conversion of heme to biliverdin-IXα (BV-IXα) with the release of Fe^2+^ and CO, is considered to be the major enzyme responsible for CO synthesis in both animals and plants (Shekhawat and Verma, 2010). This reaction requires NADPH as an electron source and molecular oxygen (Bilban et al., 2008). Plant hemoxygenases are represented by a family of four genes, of which *HO-1* is the most highly expressed (Matsumoto et al., 2004). In plant cells, the enzymatic protein HO-1 is found in chloroplasts and mitochondria (Shekhawat and Verma, 2010; Dixit et al., 2014). Thus, the localisation of CO synthesis, as well as other GTs, coincides with the subcellular localisation of melatonin formation in plants.

The data on the mechanisms of CO signal transduction in the genetic apparatus of plant cells, as well as on its relationship with other signal mediators, are still insufficient. It is known that CO can bind to the Fe atom of the heme fragment of guanylate cyclase in animal cells, thereby activating the enzyme and the synthesis of the secondary intracellular messenger cGMP (He and He, 2014). The presence of guanylate cyclase activity and cGMP in cells has been demonstrated in a number of plant species in recent years. It has been suggested that CO, like NO (Neill et al., 2008), may affect Ca^2+^ homeostasis *via* cGMP.

There are also data in the literature suggesting a functional relationship between CO and NO. For example, enhancement of lateral root formation in rapeseed induced by CO donor treatment was dependent on NO synthesis (Cao et al., 2007). Root hair development under the action of exogenous CO was accompanied by an increase in endogenous NO (Guo et al., 2009). It was also shown that stomatal closure effect induced by exogenous CO was accompanied by an increase in NO in the closing cells and was eliminated by inhibitors of NO synthesis (Song et al., 2008). The increase in heat resistance of wheat seedlings induced by the CO donor hemin was accompanied by a transient increase in NO levels in root cells associated with NR activation (Shkliarevskyi et al., 2021). Subsequently, H_2_O_2_ generation was enhanced by activation of extracellular peroxidase. However, both the effect of increased NO and the effect of increased H_2_O_2_ were supressed by various Ca^2+^ antagonists. It has been suggested that Ca^2+^ and NO are upstream of ROS in the signalling chain when heat resistance of wheat seedlings is induced by CO (Shkliarevskyi et al., 2020; 2021).

The relationship between CO and melatonin in plants has not been experimentally confirmed until recently. A review by Wang et al. (2022a) suggested a possible effect of melatonin on *HO-1* gene expression by analogy with the existence of such a mechanism in animal cells. More recently, the involvement of CO as a signalling mediator in melatonin-induced drought tolerance in tomato was investigated (Liu et al., 2024). It was shown that melatonin-induced alleviation of the growth inhibitory effects of drought was eliminated by the CO scavenger hemoglobin. Hemoglobin also eliminated the beneficial effect of melatonin on the expression of several genes related to chlorophyll and heme synthesis. Thus, based on the inhibitor analysis data, the authors suggest that CO is involved in the realisation of the stress-protective effect of melatonin on plants under drought. However, there are no direct experimental evidence confirming the effect of melatonin on *HO-1* gene expression and activity of this enzyme in plants. The connection of CO as a possible mediator in the realisation of melatonin action with other signalling molecules (ROS, NO, Ca^2+^ ions), which, as mentioned above, are involved in the signal transduction of CO when it induces stress-protective reactions in plants, remains unexplored. The potential effect of CO on melatonin synthesis in plants also remains unexplored. However, it has been shown in animal models that CO can stimulate pineal gland cells to produce melatonin (Romerowicz-Misielak et al., 2018).

## Conclusion and prospects

Melatonin is now considered a novel plant hormone and a promising regulator of plant growth and adaptation (Aghdam, and Arnao, 2024; Arnao et al., 2022). At the same time, as the authors of a recent review wittily pointed out (Aghdam and Arnao, 2024), only a decade and a half of intensive research has moved phytomelatonin from being studied as a key player in intracellular signalling to being a player in the global horticulture market. The number of articles on phytomelatonin in leading journals has increased from 33 in 2007 to over 500 in 2023 (Aghdam and Arnao, 2024).

The accumulated data show that melatonin exerts its stress-protective effect mainly through its involvement in the general signalling network of plant cells (Murch and Erland, 2021). Data have been obtained indicating the ability of melatonin to influence the state of Ca^2+^ channels, and the melatonin-induced increase in Ca^2+^ influx into the cytosol from the extracellular space and intracellular compartments may be one of the first stages of its involvement in the signalling network. Equally important appears to be the involvement of melatonin in ROS signalling. Despite the potent direct antioxidant effect of melatonin, its exogenous entry into plant cells results in a transient increase in ROS generation, which is likely primarily associated with the activation of NADPH oxidase.

The involvement of GTs in plant cell signalling and adaptation to various stresses has become one of the main directions in experimental plant biology, along with the topic of melatonin (Yao et al., 2019; Kolupaev et al., 2022). However, it is only in the last few years that knowledge has begun to accumulate, which allows us to build, at least in a general way, models of the functional interaction between melatonin and GTs. Thus, in a review published two years ago (Wang et al., 2022a), the discussion of possible functional relationships between melatonin and GTs in plant cells was mainly based on analogies with the mechanisms of their interaction in animal cells. At present, experimental data have been obtained indicating that for the manifestation of many stress-protective effects of melatonin it is necessary to enhance NO synthesis in plants. Using inhibitor analysis and partly molecular genetic methods, information on the involvement of H_2_S in the realisation of melatonin effects has also been obtained.

**Figure 6:**
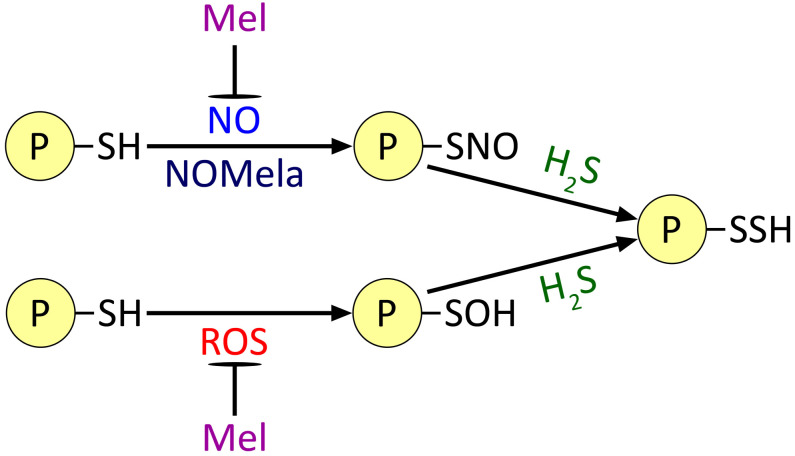
Possible effect of melatonin on the processes of post-translational modification of proteins by gasotransmitters and ROS. Mel, melatonin; NOMela, N-nitrosomelatonin; ROS, reactive oxygen species. Other explanations in the text.

At the same time, the elucidation of the functional interaction of the two most important GTs, H_2_S and NO, in the realisation of melatonin’s regulatory effects is only beginning. The ability of both GTs to induce protein PTM and the ability of melatonin to bind NO and ROS, which react with thiol groups (**Figure 6**), suggest that melatonin may act as a regulator of the PTM of at least some proteins. However, the effects of melatonin on these processes are far from clear. For example, as mentioned above, NOMela formed by the interaction of NO with melatonin may be more efficient than NO in S-nitrosation reactions of thiol groups. On the other hand, the reaction product of melatonin with ROS 2-OHM can significantly activate one of the main ROS-generating enzymes, NADPH oxidase (**Figure 3**). Considering that groups previously subjected to S-nitrosation or oxidation are involved in persulfidation reactions by H_2_S (Aroca et al., 2021), it is conceivable that melatonin may also play a prominent role in modulating the process of protein persulfidation. The hypothetical pathways outlined above only consider the direct interaction of melatonin with PTM inducers. However, there is no doubt that these *in vivo* processes are superimposed by the involvement of melatonin in the general signalling network and, as a consequence, its influence on the gene expression of many proteins. Unfortunately, the regulatory functions of melatonin as an agent potentially affecting protein PTMs involving ROS, NO and H_2_S remain largely unexplored. Individual examples of proteins whose state is affected by melatonin and its derivatives (NADPH oxidase and glyceraldehyde-3-phosphate dehydrogenase) have been given above. On the other hand, the functional interaction of NO and H_2_S GTs in protein PTM is better understood and there are considerably more examples of proteins regulated by them, including in a competitive manner (for reviews, see Arora and Bhatla, 2015; Correa-Aragunde et al., 2015; Corpas et al., 2019a, 2019; Mishra et al., 2021; Kolupaev et al., 2023b). Thus, a detailed study of the effect of melatonin on the processes of protein PTM by GTs may become one of the important areas of research that can contribute to the elucidation of the mechanisms of its stress-protective action in plant cells.

Finally, there is a clear lack of experimental data on the functional interaction between melatonin and CO in plant cells. The latter is a recognised GT of plant cells, but due to the lack of cheap and available CO donors, it has not yet found practical application in crop production. Nevertheless, elucidation of the role of CO as a likely link in the physiological effects of melatonin in plants is also necessary to fully understand the mechanisms of action of this new plant hormone.
